# Critical Events throughout the Educational Career: The Effect of Grade Retention and Repetition on School-Aged Children’s Well-Being

**DOI:** 10.3390/ijerph17114012

**Published:** 2020-06-04

**Authors:** Katharina Rathmann, Katharina Loter, Theres Vockert

**Affiliations:** 1 Department of Nursing and Health Sciences, Fulda University of Applied Sciences, 36037 Fulda, Germany; theres.vockert@pg.hs-fulda.de; 2 Institute of Sociology, Martin Luther University Halle-Wittenberg, 06108 Halle (Saale), Germany; katharina.loter@soziologie.uni-halle.de

**Keywords:** well-being dynamics, grade retention and repetition, school-aged children, distributed fixed-effects, National Educational Panel Study (NEPS)

## Abstract

More than 20% of all school-aged children in Germany experience a grade retention and repetition during the educational career which is likely to affect their well-being as a central element of school success. This study aims at revealing the temporal dynamics of general and school well-being around the event of grade retention (i.e., the year when the decision to repeat a grade is taken) and the subsequent grade repetition (i.e., the repeated school year) during secondary school in Germany. Longitudinal data from the National Educational Panel Study (NEPS) is used on native students attending grades 5 through to 12 (N = 4581 from originally 273 schools). Distributed fixed-effects regressions by gender and school type have been conducted, using satisfaction with school and life as outcomes. Although retention decisions often trigger an immediate decrease in students’ well-being, there arise still benefits from this event in the short-term, middle-term and long-term, though trajectories differ by gender and school type. Overall, it is necessary to promote students´ well-being throughout their educational career, particularly in those critical periods when they face grade retention. Results highlight that tailored programs for both genders and students in different school types should be provided to foster well-being during this phase.

## 1. Introduction

The educational career of school-aged children (herein after called students) in Germany is strongly characterized by systematic transitions due to the hierarchical structure of the educational system that provides different school types [1,2]. In most cases, transitions take place as so-called normative or regular transitions, e.g., from kindergarten to primary school, from primary to lower secondary level education and then to upper secondary level or post-secondary non-tertiary education. Furthermore, there are also non-normative or irregular transitions possible, for instance, grade repetition or change of the attended school type, that are mainly due to poor school performance and might affect school careers either. Since the 1980s, grade repetition has been critically questioned, especially by teachers, and potential negative experiences of affected students have been highlighted [3]. The reasons for the need of grade repetition include mainly a lack of promotion for the upcoming school year due to a failure in achieving the basic learning objectives of a grade in several school subjects [4]. Sometimes a grade repetition is based on a voluntary repetition decision [5]. Thus, the decision to repeat a grade is made primarily conditional on low school performance [6,7]; in cases of low school performance, teachers, parents or both decide that a student has to be retained for one school year, meaning a repetition of the same school grade. Students face then a grade retention decision. Hence, the year in which the decision to repeat a grade is made, is herein after defined as the year of grade retention. The idea of grade repetition is that retained students are unlikely to make sufficient progress in the next school grade due to their low school performance, and successful progress in learning is rather unlikely in the next school grade. The year in which students repeat a grade is subsequently herein after named grade repetition. Sometimes, grade repetition serves as a targeted measure, pursuing the idea that the level of students’ learning and achievement within a school class should be rather homogeneous in order to enable “smoother learning progresses” [5,7]. In addition, grade repetition might serve as a remedial function by offering students the opportunity to make up for previous deficits in individual school subjects without the additional burden of learning new content [7]. 

One fifth of all students (20.3%) in Germany experience a grade retention and repetition during their school career [8]. In the 2016/2017 school year, 2.3% of students repeated a grade, with noticeable differences, however, among the federal states. For instance, the rate of grade retention is 1.2% in Berlin and 4% in Bavaria: Educational systems in federal states in Germany are heterogeneous and apply grade retention decision and repetition differently, mainly due to low school performances of students. In either case, a forced or voluntary decision is required, either by teachers, parents or students´ own choice [9]. An international comparison reveals that the use of grade repetition as a pedagogical measure varies greatly among countries. The repetition rate in Germany (20.3%) is above the average rate for grade repetition in OECD countries (i.e., 12.4% of all students) [10]. In comparison, data from the PISA study 2012 (the OECD’s Programme for International Student Assessment) show that in Belgium, for example, 36.1% of students were retained once during their school career while in Great Britain only 2.7% of students, and Norway does not allow grade retentions at all [10]. 

In Germany, there is a gender-specific difference in the frequency of grade repetition showing that boys more frequently experience grade repetition during their school career than girls (2.8% of boys and 1.8% of girls) [11]. Further, the rates of grade repetition also differ among school types in Germany, showing a so-called social gradient. One should note that the German school system is hierarchically structured. After four or six years of primary school (“Grundschule”) students enrol in different types of secondary school. The school type with the highest educational standards is the grammar school (“Gymnasium”), while the school type with the lowest educational standards is the general school (“Hauptschule”). Placed in between are comprehensive schools (“Gesamtschule”), intermediate schools („Realschule“), schools with different tracks of education (“Schule mit mehreren Bildungsgängen”), and schools for students with special educational needs („Förderschule“). In 2016/17, for instance, more students repeated a grade at a general school (4.7%) than at a grammar school (2.1% in 8-year programs and 2.0% in 9-year programs). Compared to repetition rates at general schools in Germany, a lower percentage of grade repetition was found in intermediate schools (4.3%) than in schools with different tracks (3.4%). 

### 1.1. Grade Retention as a Critical Event during the School Career

All kind of transitions, whether normative or non-normative, become part of a student’s biography. According to Elder [12], a biography refers to “pathways through the age-differentiated life span, to social patterns in the timing, duration, spacing, and order of events” [13] (p.2). In the biography, a distinction can be made between “transitions” and “trajectories” [12,14]. Trajectories refer to a longer period of time [12,15] while transitions are limited in time and are about coping with discontinuous changes [16]. 

Transitions can be perceived as critical events. In general, events are defined as critical if they trigger profound emotions and stress (e.g., divorce, difficult illnesses, deaths) and require adaptation (see in particular Holmes and Rahe [17] and their Social Readjustment Rating Scale; [18]). As a result of these events, previous goals and values are called into question, and new skills and behaviors are required. Critical events do not necessarily have negative consequences and the outcome depends on individuals’ (coping) resources and the cause, magnitude, and relevance of the critical event [18]. Regarding the theoretical perspective, previous research on critical life events have shown that these potential stressors may have either negative consequences for well-being (stress theory perspective), or can trigger positive responses and change (development theory [18,19]). Following the cognitive-transactional stress concept of Lazarus and Folkman [20], the stress theory states that an interplay of the event itself and the subjective perception and evaluation by individuals is decisive on whether stress arises after a critical life event. From the perspective of the development theory, however, the potential for change arising from critical events is emphasized, from which people can benefit and gain experience [18].

Both normative (i.e., regular) transitions and irregular transitions are often described as critical events in life course and school transition research [12,18]. Critical events can manifest themselves in unsuccessful school careers of students [21,22]. For example, experiencing a grade repetition usually means a change in the learning environment, as students are in a new class with other classmates than before. The same applies when students need to change their school type, for instance, to a school type with lower educational standards (i.e., a downward transition) due to their unsatisfactory school performance or school-related misconduct. Thus, grade repetitions or downward transitions go hand in hand with adaptation tasks and opportunities for development and learning [19,22]. Adaptation tasks relate to different aspects such as social aspects (e.g., loss of stable relationships with classmates and teachers), aspects of instruction (e.g., the teaching process) or organizational aspects (e.g., new schedules and teaching structures) [1,23]. 

Critical events do not only affect the individual educational biographies of students [21,23] but also their well-being [24,25]. Thus, studying critical events throughout the educational career is important because well-being at school age is regarded as a central element for success in school, besides students´ school performance [26]. 

### 1.2. Well-Being

Well-being is defined differently in many studies [27,28], mostly as a multidimensional construct that includes psychological components (e.g., psychosomatic abnormalities), affective components (e.g., school satisfaction) and cognitive components (e.g., life satisfaction) [27,29]. However, in many studies, only individual components are examined in detail [25,28].

*General well-being* is the subjective experience of adolescents “as a cognitive evaluation of one´s overall life or important domains (e.g., family, school)” [30] (p.15). It includes both cognitive (e.g., life satisfaction) and affective components (e.g., positive emotions such as enjoyment and self-esteem and negative emotions such as depression and anxiety). Studies also distinguish between mental well-being (e.g., depression, stress [31]) and emotional well-being (e.g., peer and contact problems, hyperactivity [32]). General well-being also refers to self-esteem [17] and self-reported health [33]. 

*School well-being* is also often defined as a multidimensional construct [27,33] that comprises positive emotions (e.g., school satisfaction or school enjoyment) and negative emotions (e.g., school demotivation or school disengagement) [27,34]. Studies indicate that school well-being is divided into affective and cognitive components [34]: Affective aspects refer to the subjectively experienced emotional state of students in connection with school, while cognitive aspects include the evaluation of everyday school life by students [34]. School satisfaction is a cognitive component of school well-being and refers to students´ attitudes towards school.

### 1.3. Well-Being during the School Career

Previous studies have shown that although the overall well-being of students declines during primary school [35], it is positively associated with learning success and teaching competences [36]. Even though students indicate high average values of well-being during their educational career compared to adulthood, general well-being varies according to gender, socio-economic background and the type of school attended [37,38,39,40]. 

Qualitative and quantitative research on students’ well-being has shown different gender-specific differences, depending on the indicator of well-being [35,40,41]. A study by Hascher and Hagenauer [40], for example, revealed that girls are more likely to report positive attitudes towards school compared to boys, and girls´ enjoyment of school is more pronounced. In contrast, female students are more likely to complain about psychological problems with regard to school, are more concerned about school [40], and report lower scores of health and satisfaction with life [42,43]. Boys, however, are more likely to report lower satisfaction with school [40]. Also findings on well-being trajectories over time are heterogeneous for both genders: General and school well-being declines during school time, but to a greater extent among boys than among girls [29,35,37]. 

Alongside gender as a horizontal dimension of inequality, the vertical dimension of inequality—usually the socio-economic resources of the family of origin—also plays a key role in the psychosocial and health-related development of adolescents [44]. Studies show that belonging to a lower social, educational or income status group is likely to be associated with a lower general well-being among students [38,39]. Further, the socio-economic background of families correlates with social resources and the extent of family support [45]. Inequalities in well-being usually follow a social gradient, i.e., lower socio-economic family status (low parental education, occupational status or income) is often associated with lower well-being among young people [39,46]. Previous empirical studies focused on inequalities in well-being related to the socio-economic background of parents [47]. Current studies, however, make use also of the adolescents’ own or future socio-economic status, such as the attended type of school or subjective measures of social status that are assessed by young people themselves [38,48]. The results of these studies indicate that students who attend a school type with lower educational standards (e.g., general or schools with different tracks) are more likely to report lower life satisfaction than students who attend a school type with higher educational standards (e.g., grammar schools in Germany) [37]. Regarding commitment to school and learning among students, school types other than grammar school are often attended by students who are more likely to show greater alienation with school, and where poorer teaching quality, less autonomy and less interaction among students are more often reported [32,39]. Further, students in general schools also had an increased likelihood of sick leave days and are more likely to report poor self-reported health compared to peers in grammar schools [42]. Thus, school types differ in terms of their learning milieus [2] which in turn are associated with students´ school failure and ambition as well as their general and school-related well-being [42]. Different learning environments are primarily due to institutional differences, e.g. in curricular requirements or institutional cultures [2,49]. 

### 1.4. The Effect of Transitions on Students’ Well-Being

Overall, the majority of previous studies focused on the effects of the transition from primary to secondary school (i.e. a regular normative transition, [1,23,50]). Most of those studies found a negative effect on having experienced such transition on well-being, especially for satisfaction with school, as well as the emotional and mental well-being of adolescents [50,51]. However, only a few of those effects are of a longitudinal nature, providing evidence that the effects of the transition are usually short-lived ([52]; for further findings, see [53]). Students who have problems managing the transition are more likely to report school demotivation and disengagement [54]. Manifestation of certain behavioral patterns (e.g., lower participation in lessons or motivation [23,55]) and a decrease in satisfaction with school and appreciation of individual school subjects or teachers were also observed [1,56]. In particular, findings from qualitative studies emphasize that for some students a school transition also represents a “longed-for opportunity” [57], while for other students, it is a “fear inducing threat” [57]. Moreover, the experience of a transition may also be related to the well-being of adolescents after their school time, indicating a long-term effect [50,53].

The consequence of a grade repetition has been, so far, considered primarily in relation to academic achievement in individual school subjects and student’s motivation [58,59,60]. Meta-analyses based mostly on studies from 1990s found mixed evidence on the efficacy of early grade repetition showing it rather as an ineffective intervention for later academic achievement and socioemotional adjustment [61,62]. Some studies show a steep decline in students’ motivation in the retention year and a middle-term recovery to the baseline level within two years [60]. Also, a short-term increase in performance observed after grade repetition seem to attenuate after a few years [63]. Moreover, students who have already repeated a grade once are, in general, at higher risk to experience another grade repetition later in their educational career [6] what might negatively affect the further course of their career at school [64]. Quantitative studies conclude that grade repetition would not lead to a general improvement in school performance, and is, from an economic point of view, a “waste of money invested in education” [61] (p. 117f). Qualitative studies, however, emphasize that a general rejection of grade repetition is premature [7]. Positive consequences for well-being can result from grade repetition, especially if it was based on a voluntary decision of students and their parents [7,39]. For instance, a supportive environment outside school, a high level of students´ individual educational aspiration, or a reflective examination of the causes and consequences of grade retention could help students to perceive the event as an opportunity [7,65]. 

Previous international studies on the effect of grade retention and repetition on well-being revealed heterogeneous results [6,23,65]. A study from Luxembourg found no significant changes in students´ well-being after having experienced a grade repetition [63]. Another study, based on three school districts in Texas, showed that well-being of retained students benefits from grade repetition in the short-term and middle-term compared to regularly promoted students [65]. Hyperactivity, sadness and personal withdrawal decrease in the short- and middle-term, whereas school engagement, school belongingness and average peer-rated liking increase. The latter increased only in the short-term, but showed a substantial decrease in the longer term, possibly as result of stigmatization by new classmates. [65]. 

With regard to gender, the extent to which boys and girls differ in their well-being after being retained has hardly been investigated, so far. Few studies showed that depression and internalized behavior (i.e., hyperactivity) were more frequently reported after a regular transition from primary to secondary school for girls than for boys [33,53]. The reason for grade retention and repetition is mostly linked to low school performances [4]. A qualitative study from Switzerland highlighted that girls with low school performances have lower self-esteem compared to boys [41]. Girls more often had concerns and (psychosomatic) complaints in relation to school [33,35] or school climate [42,43]. Thus, one can hypothesize that well-being among girls is more likely to be negatively affected in the year of a grade retention decision and also during and after having experienced the grade repetition. 

As in the case of gender, there is scarce evidence on the effect of grade repetition on well-being among students attending different types of school. Previous studies examined differences in well-being between school types in a more generalized manner. They showed that students in school types other than grammar school (i.e., Gymnasium), such as general schools, reported lower well-being on average than grammar school students [37,47]. In addition, the likelihood and frequencies of grade retention differ strongly between school types [11], showing that students attending other school types than grammar school experience more frequently a grade retention and repetition. Even though we observe an increase in participation in education and higher qualification in the era of educational expansion in Germany, there are still social inequalities with regard to the transition to schools with higher educational standards, because it is still linked to the social background of parents [66]. As educational aspirations of parents with students attending grammar schools are likely to be higher, it is reasonable to assume that the impact of grade retention and repetition on well-being among students in grammar schools turns out to be more negative compared to the well-being of students in other school types. In line with this, prior findings revealed that grammar school students often show stronger performance orientation and educational demands [67]. Furthermore, because the frequency of experiencing grade repetition in grammar schools is the lowest, one can assume that this event may more negatively affect grammar students´ well-being. In contrast, students in general schools in Germany are more likely to have parents with a lower educational background and, thus, will be from a lower socio-economic background compared to students from grammar schools [2]. Intermediate schools and schools with different tracks are more heterogeneous in the composition of students with regard to these characteristics [2]. In addition, school types other than grammar schools are more often confronted with a classroom climate that is less conducive to learning [2] and attended by students from more heterogeneous socio-economic backgrounds, which in turn has an impact on the learning atmosphere, classroom climate and shared values such as the experience of a grade repetition. 

In sum, there are only few longitudinal studies [63,65] that allow conclusions to be drawn about the effect of grade retention decision and grade repetition on well-being, psychosocial outcomes, students’ motivation and achievement. Our study is the first longitudinal study looking at this effect in the German secondary educational system with regard to students’ overall and school-related well-being as outcome. Due to the high rate of grade retention and repetition in Germany, this critical life course event requires a detailed examination.

## 2. Research Objectives and Hypotheses 

### 2.1. Objectives

By the use of longitudinal data from the National Educational Panel Study (NEPS), our study aims at providing counterfactual evidence on the effect of having experienced grade retention and repetition on overall and school-related well-being of native students, differentiating by students´ socio-demographic characteristics (i.e., gender and the attended school type as a proxy information for the socio-economic background). We focus on the following research questions:

1. What is the sample percentage of students who experienced a grade retention decision and grade repetition over the secondary school career according to different socio-demographic and economic background characteristics, i.e., gender and the attended type of school?
2. What is the overall pattern of temporal dynamics of students’ well-being around the event of grade retention decision and grade repetition?
3. What are the group-specific patterns of temporal dynamics of students’ well-being (i.e., by gender and the attended type of school) around the event of grade retention decision and grade repetition?

### 2.2. Hypotheses

Based on the theoretical background and previous empirical evidence, the following hypotheses are derived in order to answer the above-mentioned research questions. First, in a descriptive manner:

**H1a** **(Hypotheses 1a):** We assume that boys experience more frequently a grade retention decision and grade repetition compared to girls.

**H1b** **(Hypotheses 1b):** We assume that the frequency of having experienced a grade retention and grade repetition follows a social gradient, indicating that students attending school types other than grammar schools experience grade retention and grade repetition more frequently.

Second, this study addresses students’ well-being dynamics around the event of grade retention decision and grade repetition. As this study examines well-being changes in the year when a grade retention decision has taken place and in subsequent years (compared to secondary school years before the retention decision, i.e., baseline level), we are able to provide a more detailed picture of well-being throughout this critical period in the secondary school career:

**H2** **(Hypotheses 2):** We assume that well-being declines in the year of grade retention decision and continues to decline over the year when the grade repetition takes place. However, according to the results of other studies mentioned above, these effects should attenuate in the subsequent years after grade repetition, indicating that students´ well-being rises and return back to the initial level.

Third, the following hypotheses address students’ well-being dynamics around the event of grade retention decision and grade repetition depending on gender.

**H3a** **(Hypotheses 3a):** According to previous findings, girls are more likely to show lower levels of well-being than boys after a regular (i.e., normative) transition from primary to secondary school. Thus, we assume that well-being of girls is likely to decrease in the year of grade retention decision and repetition because they are more concerned about school; this negative effect should attenuate in the subsequent years.

**H3b** **(Hypotheses 3b):** For boys, we expect that well-being does not change in the year of grade retention decision and grade repetition, because they do not care as much about school as girls and experience a grade repetition more frequently. Male students may not perceive a grade repetition in terms of a critical event that could affect their well-being.

Fourth, regarding well-being dynamics around the event of grade retention decision and grade repetition of students attending different school types previous evidence is very scarce. Therefore, it is hardly possible to formulate unambiguous research hypotheses:

**H3c** **(Hypotheses 3c):** We assume that well-being of students attending general schools (i.e., Hauptschule) and intermediate schools (i.e., Realschule) does not significantly change during the years of grade retention decision and grade repetition, as students in those school types are more likely to perceive those events as less critical for their well-being. This might be due to the generally higher frequency of experiencing a grade retention decision and grade repetition in these types of school.

**H3d** **(Hypotheses 3d):** We assume that well-being of students attending schools with different tracks of education (i.e., Schulen mit mehreren Bildungsgängen) declines in the years of grade retention decision and grade repetition. According to official data in Germany, students attending this type of school are less often confronted with grade repetitions as in general schools (e.g., because the change of school track is possible within the same school). Aside from that, the decline in well-being in the years of grade retention and repetition may be due to the heterogeneous composition of students in this kind of schools (i.e., socio-economic background, aspirations and educational orientation) and the general risk of being downgraded to a school track with lower educational standards. As the percentage of grade retention is rather low in schools with different tracks, the experience of this event could be perceived as an individual school failure which might negatively affect students´ well-being.

**H3e** **(Hypotheses 3e):** For students in grammar schools (i.e., Gymnasium) the lowest percentages of grade retentions have been found. We hypothesize that due to predominantly higher educational requirements, aspirations and pressure to perform, grade retention decision is likely to be experienced as school failure that might provoke a well-being shock and self-doubts among students. Hence, grammar school students are likely to experience a significant well-being decline in the year of grade retention decision and a subsequent slow recovery to the initial well-being level.

## 3. Material and Methods

### 3.1. Data

The National Educational Panel Study (NEPS) is carried out by the Leibniz Institute for Educational Research (LIfBi) at the University of Bamberg in Germany. NEPS focuses on educational processes across the life span and is part of the Framework Program for the Promotion of Empirical Educational Research funded by the German Federal Ministry of Education and Research (BMBF). NEPS started in 2010 with six cohorts (SC1: Newborn, SC2: Kindergarten children, SC3: Grade 5 (secondary schools), SC4: Grade 9, SC5: Students in higher education, SC6: Adults) which are each revisited annually or semi-annually [68] and offer a wealth of variables, in particular in the area of competence development, learning environments, educational decisions, migration background and educational returns. NEPS provides detailed longitudinal information on the German education landscape. In addition to students, survey respondents consist of class, mathematics and German language teachers as well as parents of the participating students. NEPS survey instruments were previously submitted to the respective ministries of education of the sixteen German federal states, checked and approved. NEPS works closely with the responsible data protection officers of the federal states to ensure strict compliance with the statutory data protection provisions. 

### 3.2. Sample Selection

The present study focuses on well-being trajectories of secondary students in Germany (SC3: starting with grade 5, doi:10.5157/NEPS:SC3:9.0.0). The analyses are based on nine NEPS waves from 2010/11 to 2017/2018. The final analytical sample consists of N = 4581 students from originally 273 schools in Germany.

**Figure 1:**
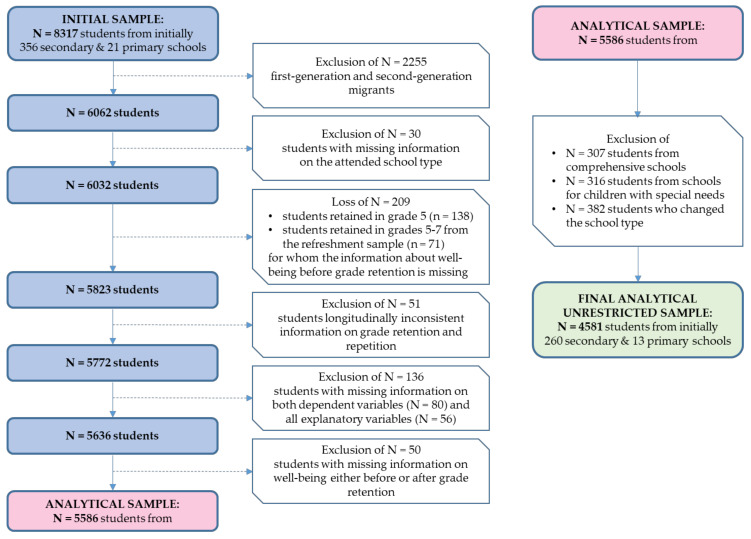
Flowchart of Analytical Sample Selection Process.

The sample selection was conducted in several steps, beginning with all available data on N = 8317 secondary students (Figure 1). First, with regard to previous findings on educational success of particular groups of migrants in Germany (with Vietnamese and Korean children as the most successful groups) and several theoretical reasons (e.g., heterogeneous parental aspirations and socialization practices, heterogeneous cultural backgrounds of children of migrants, and, primarily, their disparate patterns of adaptation to the German school system [69,70]), first-generation and second-generation migrants were excluded from the sample (N = 2255). Second, we dropped students with missing information on the attended school type (N = 30). Third, because valid information on well-being from the time before grade retention was indispensable for our analyses, and we neither have it for students from the initial sample in grade 5 (first observed wave) nor for students from the refreshment sample in grades 5 to 7 (with wave 3 as the first observed wave), we lost all observed events of grade retention and repetition that occurred in grade 5 (N = 138) and all that occurred in the refreshment sample in grades 5 to 7 (N = 71). Fourth, we identified and excluded students who reported longitudinally inconsistent information on grade retention and repetition (N = 51). Fifth, we excluded those with missing information on both dependent variables (N = 80) and all explanatory variables (N = 56). Finally, and sixth, we reduced our sample to those who reported at least two well-being values, one before and one after the event of grade retention and repetition, and ended up with a sample of N = 5586 secondary students.

From these 5586 students, N = 307 were attending comprehensive schools and N = 316 schools for children with special needs. Because the overall number of grade retentions and repetitions at those two types of school were negligibly low (N = 17 and N = 0 respectively), they cannot be considered in further analyses (N = 4963). The last group excluded from the sample were students who changed school type during their secondary school career (N = 382) reporting the highest rate of grade retentions and repetitions (19.6%; N = 75). On the one hand, in this group, well-being dynamics around grade retention and repetition are very heterogeneous with regard to downward and upward educational mobility, i.e., including not only students facing downward transfer to schools with lower demands after being retained, but also students facing upward transfer to schools with higher demands before being retained. On the other hand, due to the temporal complexity of multiple (often simultaneous) transitions in this group (to a new school, new school type and new class), causal dependencies can hardly be disentangled without implementing several additional theoretical and methodological assumptions.

### 3.3. Students who Experienced Grade Retention and Repetition and the Comparison Group

The final unrestricted analytical sample consists of N = 4581 native students enrolled in general, intermediate, grammar schools and schools with different tracks of education. From these students, N = 487 students experienced grade retention from grade 6 onwards. The respective comparison group of those who did not experience a grade retention up to grade 12 consists of N = 4094 students.

Because inadequate or unsatisfactory marks on the school certificate are, in Germany, one of the most important criteria for not being promoted to the next grade, we created a restricted analytical subsample reduced to those students who had ever had at least one sufficient (grade “4”), inadequate (grade “5”) or unsatisfactory grade (grade “6”) on their half-year or annual school certificate in any school subject surveyed by NEPS (i.e., mathematics, German, physics, chemistry, biology, natural sciences). This subsample consists of N = 3441 students who have ever been potentially at risk for grade repetition with regard to their marks on the school certificate. 

**Figure 2:**
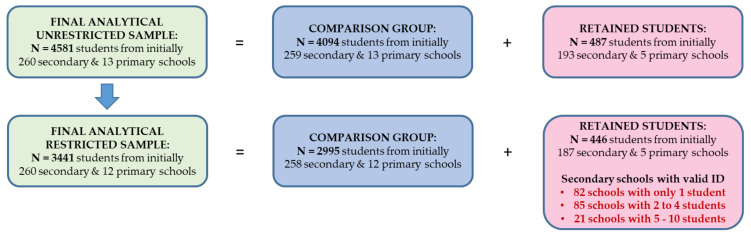
Comparison of the Unrestricted and Restricted Sample.

From these students, N = 446 experienced grade retention and repetition, a number that slightly differs from the number of grade retentions in the overall sample (N = 487). Indeed, we identified N = 41 students who experienced grade retention despite adequate school marks, mostly from intermediate (N = 12) and grammar (N = 21) schools. Possible reasons for this could be refusing school, illness, family issues, missing data or socially desirable, biased responses on school marks. Unfortunately, due to the lack of respective items, we cannot distinguish between mandatory and voluntary grade repetition, the latter with the aim of improving school marks (Figure 2).

**Figure 3:**
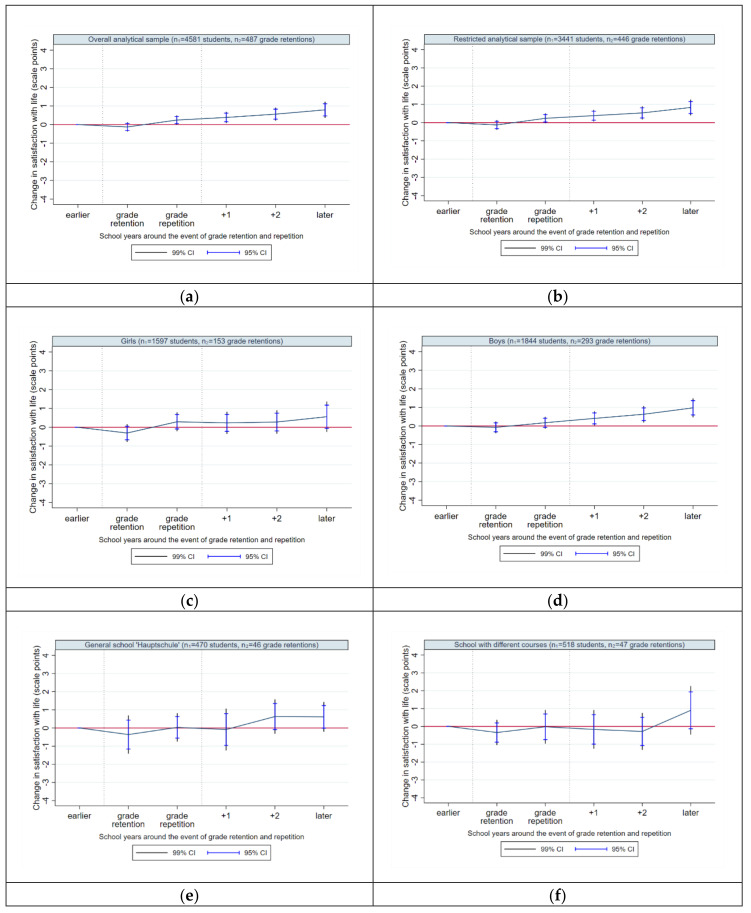

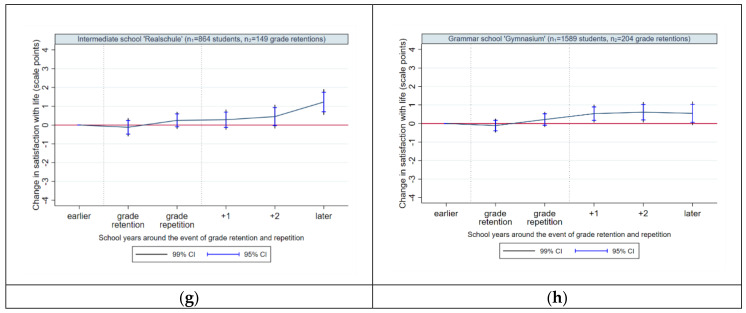
Distributed Fixed-Effects for Satisfaction with Life: Overall, by Gender and Attended School Type.

**Figure 4:**
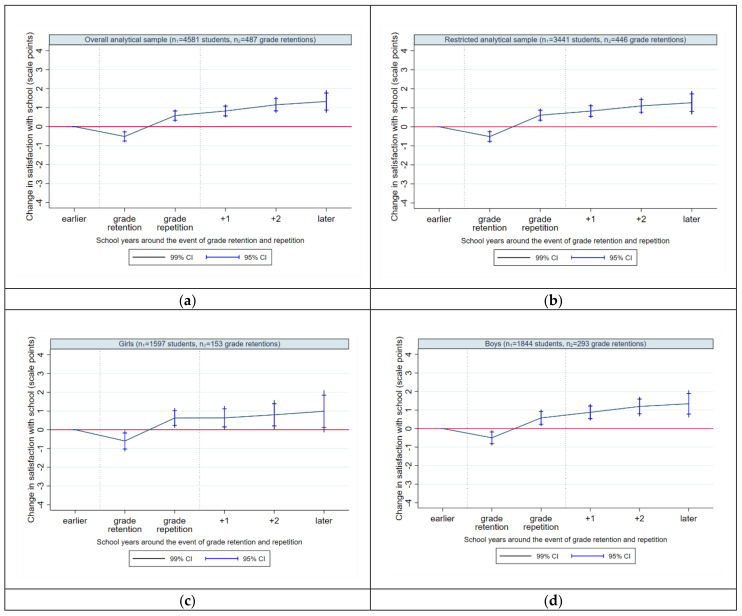

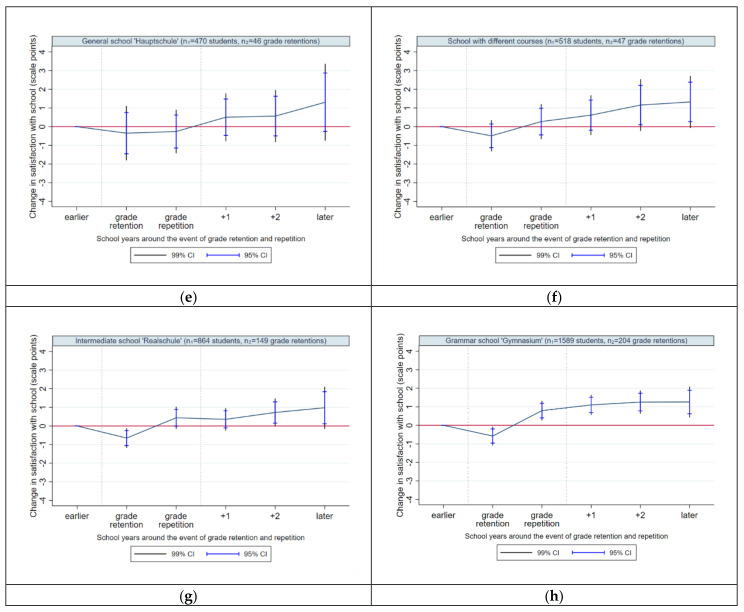
Distributed Fixed-Effects for Satisfaction with School: Overall, by Gender and Attended School Type.

The regression results for both restricted and unrestricted samples are similar (Table 2 and Table 3, Models 1 and 2, Figure 3a,b and Figure 4a,b). Because the restricted sample constitutes a substantially better comparison group than the unrestricted one, for further regression analyses we will proceed with the restricted sample of N = 3441 students, of whom N = 446 experienced grade retention and repetition and N = 2995 did not.

### 3.4. Indicators

#### 3.4.1. Dependent Variables

We focus on two student’s well-being aspects: 1) satisfaction with life and 2) satisfaction with school situation with nine available measures each, starting at wave 1 in 2010/11 (for the refreshment sample at wave 3 in 2012/13 respectively). Both outcomes are coded 0–10: “0” completely dissatisfied and “10” completely satisfied; the correlation between them equals 0.50 in the analytical sample. 

We use satisfaction with life—"How satisfied are you currently with your life in general?”—as a proxy for general well-being [71]. The individual measurement of life satisfaction has been shown to behave similarly to the psychometrically determined Satisfaction With Life Scale [72] and has been proven to be an important indicator for the well-being of adolescents [33]. 

School well-being is measured in NEPS with the question “How satisfied are you with your school situation?”. It has been shown that satisfaction with school is a reliable indicator of the successful or unsuccessful educational integration of students [73].

#### 3.4.2. Group-Specific Variables

In order to reveal group-specific well-being trajectories around the event of grade retention and repetition, we split students into time-constant distinct groups by gender and school type.

The individual school type corresponds to the school type the student was enrolled in over the observational period. We aggregated all available information about the attended school type for each student in each wave, relying mainly on sampling information, and counted the episodes. For students from the unrestricted sample who were enrolled over time constantly in one school type, we created a time-invariant group variable with: N = 507 students at general schools (with n = 47 grade retentions, retention rate 9.3%), N = 657 students at schools with different tracks (n = 54 grade retentions, rate 8.2%), N = 1051 students at intermediate schools (n = 161 grade retentions, rate 15.3%) and N = 2366 students at grammar schools (n = 225 grade retentions, rate 9.5%). Furthermore, we count N = 2218 female (n = 179 retentions, rate 8.1%) and N = 2363 male students (n = 308 retentions, rate 13.0%) in the overall sample. 

In the restricted sample of lower achieving students, the sample sizes look as follows:

- N = 1597 female students with n = 153 grade retentions (9.6%)
- N = 1844 male students with n = 293 grade retentions (15.9%)
- N = 470 students at general schools with n = 46 grade retentions (9.8%)
- N = 518 students at schools with different tracks with n = 47 grade retentions (9.1%)
- N = 864 students at intermediate schools with n = 149 grade retentions (17.2%)
- N = 1589 students at grammar schools with n = 204 grade retentions (12.8%)

#### 3.4.3. Event Time Dummies

In order to investigate changes in well-being around the event of grade retention and repetition, an event-centered time axis using time dummy variables was created. 

The reference category, i.e., the first time dummy corresponds with the time before retention decision (maximum 7 to 1 year before) and includes well-being observations of all N = 3441 students from the restricted sample (these with transition and these without transition). 

All further time dummies refer solely to students who experienced grade retention (N = 446), i.e., for whom the time is “running”. The second time dummy refers to the year of the retention decision, the third one the year of grade repetition. The subsequent three time-dummies correspond with the short-term (+1), medium-term (+2) and long-term effect of grade retention (+3 to +5, coded “later”). It is guaranteed that all N = 446 students were observed at baseline and at least one another time point after grade retention. 

#### 3.4.4. Time-Varying Confounders

We carefully decided to include only four substantively important time-varying confounders that are known from previous studies to be associated with changes in well-being around the event of grade retention and repetition. We include (1) linear, quadratic and cubic mean-centered student’s age; terms that capture both a non-linear declining trajectory of well-being over time and age-specific abilities to cope with the event. Furthermore, we incorporate (2) student’s individual self-rated health (Question: “How would you generally describe your state of health?”, coded: “0” very bad and “4” very good) that is associated with both changes in well-being and school attendance (e.g., with chronic absenteeism also related to poverty or poor parental health) [74], (3) a dummy variable for a successful transition to vocational training which for some students is a (positive) part of a recovery process after the event (coded: “0” not in training and “1” in training) and (4) a dummy variable for irrevocable dropouts (coded: “0” if a student remained in the sample at wave 9 and “1” for the last wave a student was observed at and dropped out, irrevocably) with the aim to account for potentially nonignorable missingness in trajectories of well-being. A further substantively important confounder is parental support in school matters; unfortunately, the data on these items are only of cross-sectional nature, collected at waves 4 and 9 (including a great amount of missings by design at wave 9).

Waves 4 and 7 were most affected by irrevocable dropout with respectively N = 116 and N = 162 students (i.e., overall N = 278) from all N = 564 dropouts in waves 1 to 8. Only N = 39 of them experienced grade retention and repetition. The well-being average values of those who dropped out were 8.07 for satisfaction with life and 7.16 for satisfaction with school at the last available wave. 

### 3.5. Statistical Analysis

We estimated distributed fixed-effects (FE) regression models [75] for student’s well-being by gender and school type, including all four confounders each, with age in a cubic specification. The “within” estimator compares the average well-being from baseline (time before retention decision) with the average well-being in each particular time dummy. The analysis model equation on well-being (WB) is presented below (see also [76]):

(1) $$WB_{it}=\alpha_{i}+\beta_{retention}D_{ret,it}+\beta_{repetition}D_{rep,it}+\beta_{+1}D_{+1,it}+\beta_{+2}D_{+2,it}+\beta_{later}D_{later,it}+\beta^{\prime }X_{it}+\varepsilon_{it}$$

where $D_{ret,it}$ to $D_{later,it}$ are time dummies, $X_{it}$ is a vector of time-varying confounders and $D_{earlier,it}$ (not shown in the equation) is the omitted reference category (baseline). 

**Figure A1:**
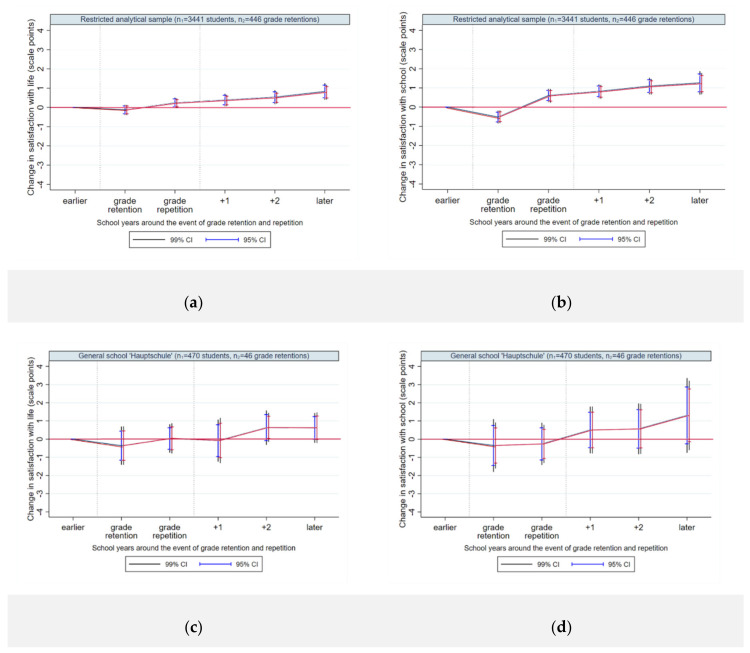
Model Comparison for Testing the Non-Independence of Observations.

All regression models were estimated with the xtreg-command in Stata (Version 16.0) with the option vce(cluster ID_t) for panel-robust standard errors. In our particular case of retained students who change their classes in the year of grade repetition (and thus, students with changing class IDs), the possibilities of making use of the nested structure of the data (in terms of students nested in classes [77]) are very limited. Unfortunately, NEPS school IDs refer solely to sampled schools from wave 1 (for refreshment sample from wave 3) and are set as missing after school change or individual tracing, e.g., due to school refusal. In longitudinal manner, accounting for school IDs would lead to an essential loss of observations at higher panel waves and thorough exclusion of students attending schools in Berlin and Brandenburg from the analytical sample (in both federal states, primary school lasts six instead of four years and the valid school IDs refer to primary school and not to secondary school). Including a comparison group to the analysis is important with regard to the estimation of the confounders, however, it should be stressed that only the group of retained students contribute to the fixed-effects estimation [78]. In the retention group, N = 446 retained students are nested in 187 secondary schools (for N = 5 students we know only the ID of the primary school). More than 40% of schools (n = 82) count only one retained student (Figure 2). Thus, in our case, the use of multilevel modeling would be very limited due to data sparseness, i.e., a small number of retained students nested in schools [79]. In the Appendix A, we provide robustness checks on our results accounting for the nested data structure. For this purpose, we apply random-effect-within-between three-level models (REWB) [80] with observations (i) nested in students who are (ii) nested in schools, finding no indication of the necessity to account for non-independence of observations (Figure A1).

## 4. Results

### 4.1. Descriptive Results

Table 1 shows the composition of the restricted sample for 1) the comparison group (N = 2995) at the first observed wave and 2) for retained students (N = 446) at both the first and last observed wave before grade retention. At the first observed wave, students from both groups were on average 11 to 12 years old and rated their health in a similar good manner; for retained students, however, the average well-being values were slightly lower than for the comparison group (average value of life satisfaction of 7.93 in the comparison group versus 7.59 in the retained group; for school satisfaction respectively 7.38 versus 6.87). The descriptive results in Table 1 indicate the highest well-being values in the comparison group and the lowest values in the group of retained students at the last wave before grade retention. For retained students, both life satisfaction and school satisfaction decline respectively from 7.59 to 7.17 and from 6.87 to 5.91 over time prior to grade retention. 

While the comparison group is almost equally composed of 52% male and 48% female students, boys predominate in the grade retention group (66% versus 34%). Whereas in the grade retention group, girls reported higher well-being values than boys at the first observed wave, the tendency changed for life satisfaction at the last observed wave before retention (6.91 among girls versus 7.30 among boys for life satisfaction; 6.21 among girls versus 5.75 among boys for school satisfaction).

The percentage distribution of the attended school type is quite similar between the comparison and the retained group. The comparison group assembles 46% of grammar school students, followed by intermediate school students (24%), students enrolled in schools with different tracks (16%) and general school students (14%). The order remains the same in the group of retained students with grammar school students as the most frequent group (46%) and general school students as the less frequent group (10%). The highest average life and school satisfaction among all school types were reported by grammar school students, both in the comparison group (8.27 and 7.81 respectively) and at the first observed wave before retention (7.81 and 7.06 respectively). In contrast, general school students and students at schools with different tracks reported the lowest well-being values (with the lowest life satisfaction value of 7.52 and school satisfaction value of 7.00 in the comparison group). At the last wave before grade retention, grammar schools slipped to second place and were overtaken by general schools with regard to life satisfaction (7.37 versus 7.25) and by intermediate schools with regard to satisfaction with school (6.19 versus 5.89). Again, students at schools with different tracks reported the lowest average well-being at the last wave before grade retention and repetition (6.81 for life satisfaction and 5.40 for school satisfaction). Most grade retentions occurred in the third and subsequent years after attending secondary school education, i.e., from grade 8 on.

### 4.2. Results from Fixed-Effects Regressions

#### 4.2.1. Satisfaction with Life

In order to graphically illustrate our results from fixed-effects regressions, we provide eight plots for satisfaction with life: for unrestricted and restricted analytical sample (Figure 3a,b), for female and male students (Figure 3c,d), and different school types (Figure 3e,h). The X-axis corresponds with the time dummies and represents time before, during and after the event of grade retention and repetition. The Y-axis shows the amount of change in students´ well-being (scale points) compared to the baseline level (i.e., compared to the average overall well-being level prior to grade retention). The pictured confidence intervals refer to 99% and 95% level of significance.

The overall life satisfaction (Figure 3b, Table 2: Model 2) is shown to deteriorate by 0.13 scale points in the year of retention decision (i.e., when students learn they will not get a promotion to the next grade), though this effect is not significant. In the year of grade repetition (i.e., when students repeat the grade in a new class), life satisfaction increases by 0.24 scale points compared to the baseline, though missing the threshold of 1% significance level. Hence, grade retention and repetition do not affect life satisfaction in an immediate significant way. However, from the first year after grade retention (starting at +1), life satisfaction starts to improve continuously and increases in the middle and long term to become highly significant up to 0.53 scale points (at +2) and up to 0.83 scale points later on (at +3/ +5) compared to baseline. Thus, retention decision and the subsequent repetition do not seem to have any impact on satisfaction with life in an immediate way. Though, from a middle-term and long-term perspective, the experience of grade retention and repetition leads to a highly significant improvement of well-being of students enrolled in secondary schools.

The gender-specific results indicate different time paths for girls (Figure 3c, Table 2: Model 3) and boys (Figure 3d, Table 2: Model 4). Similar for both groups is the result that neither retention decision nor grade repetition do affect life satisfaction in an immediate way. Male students seem to benefit significantly from grade retention already from the first year after grade repetition, by experiencing a significant improvement in life satisfaction in the short-, middle- and long-term. In contrast, life satisfaction of female students starts to improve slowly after grade repetition, however this change is not significant over time compared to the baseline. 

Looking at the school type-specific trajectories of life satisfaction, we do not observe any significant (positive or negative) change in life satisfaction in the years of retention decision and grade repetition compared to the baseline for any examined school type. For students enrolled in schools with lower educational standards (general schools and schools with different tracks), the experience of grade retention and repetition does not have any significant impact on life satisfaction at any time point after the event (Figure 3e,f, Table 2: Model 5 and 6). For intermediate and grammar schools, a temporal increase in life satisfaction is observed: Intermediate school students (Figure 3g, Table 2: Model 7) seem to take a significant advantage from grade retention and repetition in the long-term, whereas grammar school students (Figure 3h, Table 2: Model 8) in particular in the short- and middle-term.

#### 4.2.2. Satisfaction with School

With regard to school satisfaction, we provide (as for life satisfaction) further eight plots: For unrestricted and restricted sample (Figure 4a,b), for female and male students (Figure 4c,d), and for different school types (Figure 4e,h). The overall school satisfaction (Figure 4b, Table 3: Model 2) is shown to deteriorate by 0.52 scale points in the year of retention decision (i.e., when students learn they will not get a promotion to the next grade). Unlike the effect for life satisfaction, this effect is highly significant. In the year of grade repetition (i.e., when students repeat the grade in a new class), life satisfaction increases significantly, up to 0.61 scale points compared to the baseline (and by 1.13 scale points compared to the year of retention). Therefore, we observe a pattern of a dynamic change of school well-being within one year since the retention decision, starting with a temporary negative shock and evolving into an immediate well-being improvement. In the subsequent school years, the average school satisfaction increases continuously to the level of 1.26 scale points (at +3/+5). Thus, although the retention decision causes an immediate drop in school satisfaction, it normalizes and increases rapidly within a year, and improves continuously in the short-, middle- and long-term, indicating significant well-being gains.

The gender-specific results show similar trajectories of school satisfaction for girls (Figure 4c, Table 3: Model 3) and boys (Figure 4d, Table 3: Model 4), in line with its overall trajectory (see again Figure 4b). However, the pattern of improvement is faster (as it is for life satisfaction) and more concise for male students both in the short-term, middle-term and long-term. For female students, the long-term effect is missing the threshold of 1% significance. 

The school type-specific trajectories of school satisfaction yield a heterogeneous picture. For general school students (similar to the results for life satisfaction), grade retention and repetition do not affect satisfaction with school significantly at any observed time point (Figure 4e, Table 3: Model 5). For intermediate and grammar school students (Figure 4g,h, Table 3: Model 7 and 8), the retention decision implies a significant immediate decrease in school satisfaction followed, however, by an immediate recovery to the baseline level (intermediate schools) or an immediate improvement significantly above the baseline level (grammar schools). We show again that the negative impact of grade retention and repetition is only of an immediate nature. Students enrolled in schools with different tracks (Figure 4f, Table 3: Model 6) and intermediate schools (Figure 4g, Table 3: Model 7) seem to benefit from grade retention and repetition only in the long-term; yet, the effects have to be regarded with suspicion since they are missing the threshold of 1% level of significance. In contrast, grammar school students (Figure 4h, Table 3: Model 8) are the only school type that benefits from the experience of grade retention and repetition in the short-term, middle-term and long-term.

#### 4.2.3. Effects of Time-Varying Confounders

The effects of all four time-varying confounders reveal a homogenous pattern over our fixed-effects models. First, except for general school students, all estimated age terms were highly significant (with a negative linear, positive quadratic and negative cubic effect), indicating an L-shaped trajectory of school and general well-being over time. Second, the effects of self-rated health were positive and highly significant in all models, ranging from 0.50 to 0.66 for life satisfaction and from 0.41 to 0.54 for school satisfaction. Third, being in vocational training influences life satisfaction in a positive way (except for general school students); the effect varied between 0.32 for intermediate and 0.83 for female students. The same pattern applied to school satisfaction with the highest positive effect of 1.78 for grammar school students. Fourth, the effect of irrevocable dropout was significant at 1% level only for life satisfaction in the overall sample and for male students. Those students who irrevocably dropped out reported higher general well-being scores compared to those who remained in the sample.

Unfortunately, due to scarce longitudinal information on critical family events that are known to affect children’s well-being in a tremendous way, we are not able to account for separation, divorce or death of parents in our analyses. 

## 5. Discussion

To our best knowledge, this is the first longitudinal study anchored in the German educational system that examines the impact of grade retention decision and grade repetition on the overall and school-related well-being of native students at secondary schools. By using fixed-effects regression models that eliminate time-invariant intra-individual unobserved heterogeneity, it was possible to examine well-being changes around the event of grade retention and repetition, i.e., when the decision of grade retention takes place, in the subsequent year of grade repetition, and the period before and after having experienced those events. The research aims of this study were as follows: First, to examine, in a descriptive manner, the sample percentage of native students who experienced a grade retention decision and grade repetition. Second, to reveal dynamics in students’ well-being around the event of grade retention and repetition in the short-term, medium-term and long-term accounting for unobserved heterogeneity within the fixed-effects approach. Third, to reveal group-specific well-being trajectories by socio-demographic characteristics, i.e., conditional on gender and the attended type of school.

### 5.1. Grade retention in Germany: Descriptive Findings

The descriptive results support our first hypothesis—H1a—assuming that male students are more frequently affected by grade retention than female students. A brief reminder: The sample percentage of grade retentions in the unrestricted sample was 13.0% for boys and 8.1% for girls (respectively, 15.9% for boys and 9.6% for girls in the restricted sample). This gender-specific pattern is consistent with previous findings [11,63]. The reason for the gender-specific imbalance might be mainly due to the lower reading skills of boys and the more rule-compliant behavior of girls [81]. Further, girls are, in general, more likely to show a stronger perception of personal responsibility for issues related to school, schoolwork and other obligations in life, and they have, on average, better school marks than boys [82,83].

Hypothesis H1b, assuming that the percentage of native students having experienced a grade retention follows a social gradient differentiated by the attended school type, has to be withdrawn. In contrast to statistics from official data [11], students in intermediate schools are in our sample most frequently affected by a grade retention and subsequent grade repetition (15.3% in the unrestricted and 17.2% in the restricted sample), followed by grammar school students (9.5% in the unrestricted and 12.8% in the restricted sample). The percentage of retained students is the lowest (8.2% in the unrestricted and 9.1% in the restricted sample) in schools with different tracks of education. The main reason for differences in these percentages might be the positive selection effect for native students being more likely to attend schools with higher educational standards compared to migrants [69,70]. Further reasons could be, on the one hand, that grade repetition has already been abolished in some school types (i.e., schools with different tracks in the Federal state of Bremen) in some federal states (i.e., Hamburg, Rhineland Palatinate or Berlin), or they only take place with exceptions. On the other hand, a voluntary grade repetition is more likely to take place in grammar schools, since these school types are more focused on high level school performances and thus, the strive for performance improvement may be stronger than in other school types. 

### 5.2. The effect of grade retention and repetition on students´ well-being: Results from fixed-effects regressions

One of the most unambiguous and important findings of our study (contrary to the assumption in hypothesis H2), is that students’ well-being benefits from a grade retention decision and repetition in the short-term, middle-term and long-term instead of being penalized by these events. Our results reveal only an immediate negative effect in the year of grade retention decision on school satisfaction (but not on life satisfaction) that, however, vanishes quickly within the subsequent years after grade repetition. Unlike previous studies, which mainly used growth curves and propensity score matching [65,84], and similar to the longitudinal study on students’ motivation by Kretschmann et al. [58], we do not find any evidence for an enduring negative impact of grade repetition on students’ well-being. From a longitudinal perspective, grade retention and repetition do not seem to be purposeless; our results indicate its effectiveness, starting already in the year of grade repetition. Even if a grade retention decision may initially be experienced by students as a critical event, it seems to be associated longitudinally with a fast adaptation to peers and circumstances in the new class as well as further developmental opportunities, e.g., an improvement in school performance [60]. The opportunities resulting from a grade retention decision and grade repetition are well expressed by the phrase “the positive lessons of loss” [18], (p. 99) which highlights that critical life events might trigger a crucial individual cognitive, emotional and behavioral transformation that offers “a second chance” in student’s life. These individual responses, however, are assumed to differ depending on gender or attended school type, which is discussed in detail in the section below.

### 5.3. Grade Retention: Gender-Specific Results

With regard to life satisfaction, hypothesis H3a assuming that girls’ general well-being decreases in the years of grade retention decision and grade repetition with a recovery in the subsequent years, can only be partially supported by our results. The same applies to H3b assuming that boys’ general well-being does not change in the year of grade retention decision and in the subsequent years. Our study shows that grade retention decision does not affect life satisfaction in an immediate way; this applies to both genders. For male students, life satisfaction does not change in the year of retention decision, begins, however, to significantly increase from the year of grade repetition. For female students the effect of grade retention on life satisfaction is not significant over time. Due to the lack of studies on gender-specific well-being dynamics around the event of grade retention and repetition, we refer to studies dedicated to normative transitions (e.g., transitions from primary to secondary school) and their impact on life satisfaction. In general, findings from prior studies are rather heterogeneous [1,31,51,85]. Girls are more likely to report higher levels of depression and internalized behavior after having experienced a transition to secondary school [33,53], whereas boys report significantly more contact and peer problems as well as problems of externalizing behavior [31,33]. Overall, the current state of research shows that boys and girls react differently to critical events in terms of transitions [31,33,53]. Although previous findings reveal an increase in contact and peer problems in boys after a normative transition [32], the negative trajectory is not apparent for boys´ life satisfaction with regard to the event of grade retention and repetition. In contrast, our results show an increase in boys’ life satisfaction in the middle-term and long-term. This might be due to the fact that boys are more at risk of repeating a grade than girls, i.e., grade retention is socially less stigmatizing for them. Further, self-esteem among boys might also be less exposed to stigma by their new classmates. Boys are less worried about school and, thus, a grade repetition is considered to be less problematic. For girls, our results show no change in life satisfaction over time compared to the baseline level prior to grade retention. This could be explained by girls’ generally higher intrinsic performance attribution [41], which is likely to be accompanied by anger about, and frustration of, not having accomplished a high level of school performance. This, in turn, affects their life satisfaction negatively, preventing its greater increase. Overall, very little is known about gender-specific coping with critical life events at school. Previous studies show that boys are more likely to focus on the present, while girls tend to worry more about the future [86]. Boys are also, in general, better at ignoring problems [87], seeking for distraction by engaging in hobbies or participating in sports [88].

With regard to satisfaction with school, hypothesis H3a for girls is partially supported by our results, hypothesis H3b for boys, however, has to be rejected. For both genders, school satisfaction decreases in the year of grade retention decision and improves continuously afterwards; this pattern is more concise for boys. As already mentioned, there is a research gap on longitudinal studies on the effect of grade retention on school satisfaction to date. The existing evidence from related studies indicates that girls report higher levels of perceived pressure and demands from school compared to boys [89]. Girls are also more at risk for school-related burnout [90] and decline in self-esteem [91]. A study from Switzerland [41] shows gender-specific differences in the impact of school marks on self-esteem, revealing a stronger negative effect of poor school marks on girls´ self-esteem compared to boys. Boys tend to show more frequently externalizing behavior due to their low school performance attribution and are more likely to explain their poor performances at school by not relating it to a possible failure by themselves, but rather to external circumstances, e.g., the demands of teachers [41]. It is known that students’ self-concept suffers from the experience of grade retention [59]. Because this seems to be more pronounced for girls than boys, a slower recovery from grade retention may be assumed for girls’ school satisfaction. Some studies show that a better school performance and school grades go hand in hand with a higher satisfaction with school [40,92]. Although previous findings stress that girls more frequently report positive attitudes towards school, more enjoyable experiences at school and higher school-related well-being compared to boys [35,41,93], what may suggest a faster recovery from repeating a grade in school satisfaction for girls, this is not fully reflected in our results. Boys not only fully recover from grade retention but also improve faster in their school well-being. 

Due to the general lack of longitudinal studies on gender-specific well-being trajectories around the event of grade retention and repetition, not to mention the lack of studies on secondary school system in Germany, while discussing our findings, we help us with studies on primary schools, e.g., by Wu et al. [65]. The study reveals that students´ mean rated liking increases in short-term after having experienced grade retention, but decreases in middle-term and long-term [65]. What we observe in our study, is rather a continuous improvement of school-related well-being for both genders. The discrepancy between (not really comparable) previous findings and our results may be additionally attributed by a possible age effect. It seems likely that students—depending on their age—have different abilities to cope with the event of grade retention and repetition. A study from Germany on the transition from primary to secondary school shows that students who change to a secondary school after 4th grade of primary school report a lower academic self-concept than students who change after 6th grade [24], e.g., in Berlin or Brandenburg. 

### 5.4. Grade Retention: School Type-Specific Results

With regard to general well-being of students attending different school types, hypothesis H3c assumed that well-being does not change around the event of grade retention and repetition for students attending general and intermediate schools. Hypothesis H3c is supported by our results for general school students, but not for intermediate school students; the latter showing a long-term increase in life satisfaction after repeating a grade. Hypothesis H3d for students at schools with different tracks has also to be withdrawn. We observe no significant change in life satisfaction after being retained for these students (as for general school students). The same applies to hypothesis H3e assuming for grammar school students a significant decline in life satisfaction in the year of grade retention with a slow recovery in subsequent years. Grammar school students report no immediate changes in general well-being in the years of grade retention and repetition, but a significant improvement in short-term and middle-term. 

To date, there is no empirical evidence on well-being trajectories around the event of grade retention and repetition conditional on the attended school type. Previous studies reveal, in general, that lower life satisfaction [43] has been reported among students in school types with lower educational standards (i.e., general schools in Germany). Studies using longitudinal data show, e.g., that students at general schools report lower life satisfaction at the beginning of grade 5 (just after the transition to secondary school) than students from other school types [37]. An explanation for this could be that at the end of primary school and the beginning of secondary school, students start to perceive differences in social composition and educational aspirations between different secondary school types [67]. This may lead to a reflection and consolidation of their educational position in the educational system [67]. For instance, students from school types with lower educational standards, e.g., general schools in Germany, are more likely to be confronted with feelings of being “left behind” and get “used to failure” due to numerous setbacks they have already experienced during their school career (so-called “failed careers” [94]), i.e., more often grade repetitions, retentions due to the lack of school readiness or being downgraded to type of school with lower educational standards and aspirations [67]. This might be the reason why, in our study, the event of grade retention and repetition does not negatively affect life satisfaction of general school students and students from schools with different tracks. A study by Kramer et al. [67] on child orientation frameworks for students at lower secondary schools find that early failure in the form of, e.g., grade retention in primary school lead to a stronger feeling of distance from school in the long run [67]. In addition, some studies emphasize a strong association between students’ well-being and their socio-economic background often approximated by the attended type of school, i.e., students with a higher socio-economic background have a higher likelihood of attending school types such as grammar schools [38,39]. 

For school satisfaction, hypothesis H3c is, again, fully supported for general school students, but not for intermediate school students; the latter showing a significant decline in school well-being in the year of grade retention. As for the case of life satisfaction, hypothesis H3d for students at schools with tracks has to be rejected. Overall, our results indicate certain similarities in well-being dynamics among general school students and students at schools with tracks (mostly general and intermediate school tracks). The level of school satisfaction of intermediate school students is similar around the event of grade retention and repetition similarly to this of grammar school students; the latter revealing, however, the most advantageous pattern that allows us to partially withdraw the hypothesis H3e. Intermediate school students and grammar school students experience a significant decrease in school satisfaction in the year of grade retention, however only the school satisfaction of grammar school students improves continuously over time. The difference in dynamics of school well-being between students at general schools (and schools with tracks), and students attending other school types might be related to less demanding learning and achievement requirements than those at other school types, often internalized by students [78]. Another explanation is that students in schools with lower educational standards find themselves in an environment in which being retained is a common and shared experience among classmates. A qualitative study on the educational orientation of students in so-called “exclusive” educational settings in Germany (i.e., grammar schools) concludes that those students are more likely to be characterized by a strong orientation towards school achievement [95]. Even if it can be assumed that a higher aspiration for school success at grammar schools [67,95] may lead to a decrease in school satisfaction around the event of grade retention and repetition, our results reveal different dynamics. Grade repetition has, contrary to hypothesis H3e, a positive immediate, short-, middle- and long-term effect on school satisfaction of students in grammar schools, maybe because those students are more likely to experience a voluntary grade repetition than students in other school types. They are also more likely to live in families with higher socio-economic background and being supported by their relatives in order to cope with critical events. A central aspect in experiencing such events is that students have to make adjustments to previously habitualised behaviors that have been interrupted by the event itself [18]. Consequently, each student has a personal evaluation of the event of grade retention and repetition, and makes an individual adjustment to this event. Whether this event is perceived to be critical in the long-term depends on social and institutional support (e.g., peers, family, teachers), as in the example of coping with a normative transition from primary to secondary school [96].

In sum, the findings of this study highlight that the event of grade retention and repetition seems to be perceived rather as an opportunity than a critical event with regard to students´ well-being, in particular for boys and grammar schools students. Despite the change of from the previous familiar class environment, grade repetition affects well-being of retained students mostly in a positive manner. As a conclusion of this study, the event of grade repetition and retention should not be classified overhasty as not having any positive effects on students´ well-being. Further longitudinal studies are essential to understand more about well-being dynamics of students in German educational system, considering, e.g., social and institutional support or regional variation among federal states in making decisions about voluntariness and efficacy of grade retention as an intervention in students’ educational career.

## 6. Limitations

One of the strengths of this study is the use of longitudinal NEPS data, applying fixed-effects regressions. For the first time in the German context, conclusions can be drawn about temporary dynamics of student’s well-being around the event of grade retention and repetition. The stratified cluster sample of NEPS allows the best possible representation of the German school landscape, one that is undistorted across school types, federal states and regions [68]. 

It should be noted that a stratified analysis by the federal state of NEPS data is only permitted under certain conditions. Due to the federal state-specific design of school system, differences in well-being observed between school types may be attributable to variances among federal states. Further studies are also encouraged to use multiple items of general and school-related well-being. From this, the multidimensional construct of well-being, not available in NEPS, can be captured more precisely and target group-specific measures can be developed.

NEPS includes data on a broad variety of student migration backgrounds. Previous studies have already shown that ethnic groups differ both in their school performance and their educational goals and aspirations [69]. Because ethnic heterogeneity is assumed to trigger heterogeneous effects on the relevance of grade retention for general and school well-being, all migrants were excluded from our analysis. Future studies are encouraged to investigate the importance of grade retention for the well-being of students with a migration background.

Lastly, due to limited data, we are not able to differentiate between involuntary and voluntary grade retentions and repetitions. Because previous studies show that the impact of grade retention for well-being might differ depending on whether retention was voluntary or whether it took place at an upper secondary level, also this needs to be examined more closely in follow-up studies. 

## 7. Conclusions

Our study highlights the experience of being retained as rather advantageous for secondary students’ well-being, except for the immediate negative effect in the year of retention decision that, however, attenuates or turns positive already in the year of grade repetition. This study shows positive short-term, middle-term and long-term effects on general- and school-related well-being, particularly for boys and students in grammar schools. The patterns are more pronounced for school well-being compared to general well-being. 

Previous evidence provides heterogeneous findings on the effects of school transitions on well-being of students. An adequate examination of the role of school transitions on student’s well-being requires a longitudinal view at particular groups of students (e.g., conditional on gender or school types) and several indicators of well-being. In our study, it is not possible to make any statements about potential benefits of grade retention for school performance. However, this study highlights that critical voices against grade retention should consider the positive effects revealed in this study, particularly on the well-being of boys and grammar school students. According to our results, grade repetition is not effective for general well-being of girls (unlike school well-being) and well-being of students attending school types other than grammar school. The general trend, though, beginning from the repetition year tends to be positive also for other school types, even if not significant.

Since there are hardly any initiatives to promote well-being of retained students, it is important for future research and interventions to review best practice examples to monitor and support them during this critical period. Previous findings suggest that teachers can provide support during periods of transition [21]. Positive findings on the impact of social and professional support by peers, teachers and parents observed during normative transitions from primary to secondary schools could also be adapted to grade retention when designing future interventions during school transitions [1,96]. Future initiatives should also consider support for students coping with grade retention and repetition [31], e.g., accompanied by teachers before and after the transition to the new class [21]. This could have a positive effect on students’ well-being since a positive assessment of an imminent transition often goes hand in hand with a positive development of well-being after a transition [85].

## Figures and Tables

**Table 1:** Sample Composition (Restricted Sample) for Students Without and with Grade Repetition.

Sample composition	Comparison Group (First Observed Wave) (N = 2995)	Retained Students (First Observed Wave) (N = 446)	Retained Students (Last Wave Before Retention) (N = 446)
Well-being (outcomes)			
Life satisfaction (LSat)	7.93 (2.30)	7.59 (2.53)	7.17 (2.25)
School satisfaction (SSat)	7.38 (2.48)	6.87 (2.72)	5.91 (2.45)
Gender			
- female	48% (N = 1444) (LSat: 7.82, SSat: 7.44)	34% (N = 153) (LSat: 7.71, SSat: 7.42)	34% (N = 153) (LSat: 6.91, SSat: 6.21)
- male	52% (N = 1551) (LSat: 8.03, SSat: 7.32)	66% (N = 293) (LSat: 7.54, SSat: 6.58)	66% (N = 293) (LSat: 7.30, SSat: 5.75)
School type (time-constant)			
- general (‘Hauptschule’)	14% (N = 424) (LSat: 7.58, SSat: 7.00)	10% (N = 46) (LSat: 7.63, SSat: 6.57)	10% (N = 46) (LSat: 7.37, SSat: 5.59)
- with different tracks (‘Schule mit mehreren Bildungsgängen’)	16% (N = 471) (LSat: 7.52, SSat: 7.00)	11% (N = 47) (LSat: 7.04, SSat: 6.79)	11% (N = 47) (LSat: 6.81, SSat: 5.40)
- intermediate (‘Realschule’)	24% (N = 715) (LSat: 7.74, SSat: 7.03)	33% (N = 149) (LSat: 7.46, SSat: 6.72)	33% (N = 149) (LSat: 7.10, SSat: 6.19)
- grammar (‘Gymnasium’)	46% (N = 1385) (LSat: 8.27, SSat: 7.81)	46% (N = 204) (LSat: 7.81, SSat: 7.06)	46% (N = 204) (LSat: 7.25, SSat: 5.89)
Time-varying confounders			
Age	11.90 (1.11)	11.62 (1.00)	13.91 (1.48)
Self-rated health	3.27 (0.74)	3.16 (0.82)	3.08 (0.84)
Vocational training	0% (N = 1)	0% (N = 0)	0% (N = 0)
Irrevocable dropout	0% (N = 11)	0% (N = 0)	0% (N = 0)
Additional information:			
Grade retention in			
- Grade 6	---	4% (N = 18)	
- Grade 7	---	13% (N = 57)	
- Grade 8	---	21% (N = 95)	
- Grade 9	---	23% (N = 104)	
- Grade 10	---	21% (N = 93)	
- Grade 11	---	18% (N = 79)	

Note: LSat: life satisfaction; SSat: school satisfaction; sample distribution unweighted, mean values (and standard deviations), and frequencies in %.

**Table 2:** Distributed Fixed-Effects for Satisfaction with Life: Overall, by Gender and Attended School Type.

Fixed-Effects Results for Life Satisfaction	Model 1 (Figure 3a) Overall Sample	Model 2 (Figure 3b) Restricted Sample	Model 3 (Figure 3c) Girls	Model 4 (Figure 3d) Boys
	$\hat{b}\;\left(\hat{SE}\right)$	$\hat{\;b}\;\left(\hat{SE}\right)$	$\hat{b}\;\left(\hat{SE}\right)$	$\hat{b}\;\left(\hat{SE}\right)$
*D1:* Grade retention	−0.13 (0.10)	−0.13 (0.10)	−0.31 (0.18)	−0.07 (0.12)
*D2:* Grade repetition	0.24 (0.10) *	0.24 (0.11) *	0.29 (0.19)	0.17 (0.13)
*D3:* +1	0.38 (0.12) ***	0.38 (0.13) **	0.23 (0.23)	0.41 (0.15) **
*D4:* +2	0.56 (0.14) ***	0.53 (0.14) ***	0.28 (0.24)	0.63 (0.17) ***
*D5:* Later	0.79 (0.17) ***	0.83 (0.17) ***	0.56 (0.31)	0.98 (0.20) ***
Age (linear)	−0.79 (0.06) ***	−0.86 (0.08) ***	−0.85 (0.11) ***	−0.85 (0.11) ***
Age (quadratic)	0.16 (0.02) ***	0.18 (0.02) ***	0.15 (0.03) ***	0.20 (0.03) ***
Age (cubic)	−0.01 (0.00) ***	−0.01 (0.00) ***	−0.01 (0.00) ***	−0.01 (0.00) ***
Self-rated health	0.55 (0.02) ***	0.59 (0.03) ***	0.61 (0.04) ***	0.55 (0.04) ***
Vocational training	0.75 (0.05) ***	0.73 (0.06) ***	0.83 (0.09) ***	0.69 (0.08) ***
Irrevocable dropout	0.27 (0.09) **	0.28 (0.10) **	0.16 (0.13)	0.40 (0.15) **
*Number of cases*				
Number of person-years	N = 29,872	N = 22,002	N = 10,337	N = 11,665
Number of individuals	N = 4581	N = 3441	N = 1597	N = 1844
R-square within	0.069	0.073	0.092	0.064
	**Model 5** **(Figure 3e)** **General School**	**Model 6** **(Figure 3f)** **School with Tracks**	**Model 7** **(Figure 3g)** **Intermediate School**	**Model 8** **(Figure 3h)** **Grammar School**
	$\hat{b}\;\left(\hat{SE}\right)$	$\hat{\;b}\;\left(\hat{SE}\right)$	$\hat{\;b}\;\left(\hat{SE}\right)$	$\hat{\;b}\;\left(\hat{SE}\right)$
*D1:* Grade retention	−0.36 (0.41)	−0.34 (0.27)	−0.12 (0.19)	−0.11 (0.14)
*D2:* Grade repetition	0.03 (0.30)	−0.02 (0.36)	0.25 (0.17)	0.22 (0.16)
*D3:* +1	−0.08 (0.45)	−0.17 (0.42)	0.28 (0.21)	0.53 (0.18) **
*D4:* +2	0.63 (0.37)	−0.28 (0.40)	0.45 (0.24)	0.61 (0.21) **
*D5:* Later	0.61 (0.32)	0.90 (0.53)	1.22 (0.26) ***	0.55 (0.25) *
Age (linear)	−0.59 (0.28) *	−1.20 (0.24) ***	−0.81 (0.18) ***	−0.84 (0.10) ***
Age (quadratic)	0.13 (0.06) *	0.27 (0.06) ***	0.17 (0.04) ***	0.16 (0.02) ***
Age (cubic)	−0.01 (0.00)	−0.02 (0.00) ***	−0.01 (0.00) ***	−0.01 (0.00) ***
Self-rated health	0.50 (0.07) ***	0.66 (0.07) ***	0.58 (0.05) ***	0.58 (0.04) ***
Vocational training	0.14 (0.16)	0.79 (0.16) ***	0.32 (0.10) ***	0.73 (0.16) ***
Irrevocable dropout	0.10 (0.27)	0.36 (0.22)	0.14 (0.16)	0.28 (0.21)
*Number of cases*				
Number of person-years	N = 2393	N = 2916	N = 5514	N = 11,179
Number of individuals	N = 470	N = 518	N = 864	N = 1589
R-square within	0.056	0.095	0.083	0.080

Notes: * *p* < 0.05. ** *p* < 0.01. *** *p* < 0.001.

**Table 3:** Distributed Fixed-Effects for Satisfaction with School: Overall, by Gender and Attended School Type.

Fixed-Effects Results for School Satisfaction	Model 1 (Figure 4a) Overall Sample	Model 2 (Figure 4b) Restricted Sample	Model 3 (Figure 4c) Girls	Model 4 (Figure 4d) Boys
	$\hat{b}\;\left(\hat{SE}\right)$	$\hat{\;b}\;\left(\hat{SE}\right)$	$\hat{b}\;\left(\hat{SE}\right)$	$\hat{b}\;\left(\hat{SE}\right)$
*D1:* Grade retention	−0.52 (0.12) ***	−0.52 (0.13) ***	−0.60 (0.22) **	−0.50 (0.16) **
*D2:* Grade repetition	0.58 (0.12) ***	0.61 (0.13) ***	0.63 (0.20) **	0.57 (0.17) ***
*D3:* +1	0.82 (0.13) ***	0.82 (0.14) ***	0.63 (0.25) **	0.88 (0.17) ***
*D4:* +2	1.15 (0.16) ***	1.10 (0.17) ***	0.80 (0.30) **	1.19 (0.20) ***
*D5:* Later	1.32 (0.23) ***	1.26 (0.24) ***	0.98 (0.44) *	1.33 (0.28) ***
Age (linear)	−0.92 (0.07) ***	−1.20 (0.09) ***	−1.16 (0.12) ***	−1.23 (0.12) ***
Age (quadratic)	0.18 (0.02) ***	0.24 (0.02) ***	0.22 (0.03) ***	0.26 (0.03) ***
Age (cubic)	−0.01 (0.00) ***	−0.01 (0.00) ***	−0.01 (0.00) ***	−0.02 (0.00) ***
Self−rated health	0.46 (0.02) ***	0.49 (0.03) ***	0.49 (0.04) ***	0.48 (0.04) ***
Vocational training	1.17 (0.07) ***	1.25 (0.08) ***	1.28 (0.12) ***	1.21 (0.11) ***
Irrevocable dropout	0.01 (0.10)	−0.00 (0.13)	0.04 (0.19)	−0.03 (0.17)
*Number of cases*				
Number of person-years	N = 29,872	N = 22,002	N = 10,337	N = 11,665
Number of individuals	N = 4581	N = 3441	N = 1597	N = 1844
R-square within	0.065	0.076	0.083	0.072
	**Model 5** **(Figure 4e)** **General School**	**Model 6** **(Figure 4f)** **School with Tracks**	**Model 7** **(Figure 4g)** **Intermediate School**	**Model 8** **(Figure 4h)** **Grammar School**
	$\hat{b}\;\left(\hat{SE}\right)$	$\hat{\;b}\;\left(\hat{SE}\right)$	$\hat{\;b}\;\left(\hat{SE}\right)$	$\hat{\;b}\;\left(\hat{SE}\right)$
*D1:* Grade retention	−0.35 (0.56)	−0.49 (0.32)	−0.65 (0.21) **	−0.58 (0.20) **
*D2:* Grade repetition	−0.26 (0.45)	0.27 (0.36)	0.44 (0.23)	0.79 (0.20) ***
*D3:* +1	0.50 (0.50)	0.62 (0.41)	0.36 (0.23)	1.10 (0.21) ***
*D4:* +2	0.56 (0.54)	1.16 (0.54) *	0.72 (0.29) *	1.25 (0.24) ***
*D5:* Later	1.31 (0.79)	1.32 (0.54) *	0.98 (0.44) *	1.26 (0.32) ***
Age (linear)	−0.67 (0.32) *	−1.42 (0.26) ***	−1.21 (0.19) ***	−1.16 (0.11) ***
Age (quadratic)	0.14 (0.07)	0.33 (0.06) ***	0.27 (0.04) ***	0.21 (0.03) ***
Age (cubic)	−0.01 (0.00)	−0.02 (0.00) ***	−0.02 (0.00) ***	−0.01 (0.00) ***
Self-rated health	0.41 (0.08) ***	0.48 (0.07) ***	0.54 (0.05) ***	0.48 (0.04) ***
Vocational training	0.53 (0.21) **	1.40 (0.18) ***	0.75 (0.13) ***	1.78 (0.24) ***
Irrevocable dropout	−0.43 (0.30)	−0.05 (0.25)	−0.31 (0.23)	0.48 (0.24) *
*Number of cases*				
Number of person-years	N = 2393	N = 2916	N = 5514	N = 11,179
Number of individuals	N = 470	N = 518	N = 864	N = 1589
R-square within	0.047	0.087	0.086	0.095

Notes: *p < 0.05. ^**^p < 0.01. ^***^p < 0.001.

## Appendix A

Additional information: Blue lines correspond to results ignoring the nested structure of the data (i.e., results presented in our manuscript; red lines correspond to results from REWB (hybrid) models accounting for the nested structure of the data. To avoid essential loss of observations at higher panel waves and thorough exclusion of students attending schools in Berlin and Brandenburg, in all cases school IDs from the first observed wave have been applied to all subsequent waves (i.e., irrespective of the possibility of school change). Exemplarily, we show models for life and school satisfaction for the restricted overall sample and for general secondary schools with the smallest sample sizes. We are assured of the robustness of all other results presented and discussed in the manuscript.
